# The Activity of Nodules of the Supernodulating Mutant Mt*_sunn_* Is not Limited by Photosynthesis under Optimal Growth Conditions

**DOI:** 10.3390/ijms15046031

**Published:** 2014-04-10

**Authors:** Ricardo A. Cabeza, Annika Lingner, Rebecca Liese, Saad Sulieman, Mehmet Senbayram, Merle Tränkner, Klaus Dittert, Joachim Schulze

**Affiliations:** 1 Department for Crop Science, Section for Plant Nutrition and Crop Physiology, Faculty of Agriculture, University of Goettingen, Carl-Sprengel-Weg 1, Goettingen 37075, Germany; E-Mails: rcabeza@u.uchile.cl (R.A.C.); alingne@agr.uni-goettingen.de (A.L.); rebecca.liese@biologie.uni-goettingen.de (R.L.); klaus.dittert@agr.uni-goettingen.de (K.D.); 2 Departamento de Ingeniería y Suelos, Facultad de Ciencias Agronómicas, Universidad de Chile, Av. Santa Rosa, La Pintana, Santiago de Chile 11315, Chile; 3 Signaling Pathway Research Unit, RIKEN Center for Sustainable Resource Science, 1-7-22, Suehiro-cho, Tsurumi, Yokohama 230-0045, Japan; E-Mail: s.sulieman@psc.riken.jp; 4 Institute for Applied Plant Nutrition, Faculty of Agriculture, University of Goettingen, Carl-Sprengel-Weg 1, Goettingen 37075, Germany; E-Mails: msenbay@uni-goettingen.de (M.S.); mtraenk@gwdg.de (M.T.)

**Keywords:** nodules, legumes, H_2_ evolution, supernodulation, *Medicago truncatula*, P-depletion, photosynthesis, elevated CO_2_

## Abstract

Legumes match the nodule number to the N demand of the plant. When a mutation in the regulatory mechanism deprives the plant of that ability, an excessive number of nodules are formed. These mutants show low productivity in the fields, mainly due to the high carbon burden caused through the necessity to supply numerous nodules. The objective of this study was to clarify whether through optimal conditions for growth and CO_2_ assimilation a higher nodule activity of a supernodulating mutant of *Medicago truncatula* (*M. truncatula*) can be induced. Several experimental approaches reveal that under the conditions of our experiments, the nitrogen fixation of the supernodulating mutant, designated as *sunn* (super numeric nodules), was not limited by photosynthesis. Higher specific nitrogen fixation activity could not be induced through short- or long-term increases in CO_2_ assimilation around shoots. Furthermore, a whole plant P depletion induced a decline in nitrogen fixation, however this decline did not occur significantly earlier in *sunn* plants, nor was it more intense compared to the wild-type. However, a distinctly different pattern of nitrogen fixation during the day/night cycles of the experiment indicates that the control of N_2_ fixing activity of the large number of nodules is an additional problem for the productivity of supernodulating mutants.

## 1. Introduction

Legumes cover up to 90% of their N demand through a root/*Rhizobia* symbiosis [1]. However, N_2_ fixation in the nodules of the roots is a costly process at a whole plant level. Estimates based on greenhouse pot experiments reveal that the carbon costs for driving nitrogenase corresponds to about ¼ of shoot dry matter at the end of a growing season [2]. It is thus understandable that legumes—by various measures—keep the nitrogen fixation at the lowest necessary level and use any alternative nitrogen form preferentially [3]. Among these mechanisms, the first and most important is to match the total nodule number to plant growth and N demand. For that purpose, legumes have evolved a molecular mechanism that involves root-shoot signaling [4]. This autoregulation of nodulation (AON) consists in short of the following steps. The Nod-factors produced by the bacteria not only induce nodulation but also the formation of xylem-mobile Clavata3/Embryo Surrounding Region-Related (CLE) peptides. These peptides travel to the shoot and bind to and activate a Leucine-rich repeat (LRR) receptor-like kinase. The receptor kinase resembles the *Arabidopsis CLAVATA1* gene [5]. The activated receptor kinase induces a not fully understood cascade of events that result in an only partially characterized compound, the so called shoot-derived inhibitor (SDI) [6]. SDI itself is transported in the phloem to the roots, inhibits nodule meristem growth and thus further nodule formation. The LRR receptor-like kinase represents a crucial step in the regulatory cascade and the root-shoot-root signaling. Legumes that carry a mutation in that gene show a so called super- or hypernodulating phenotype, forming the manifold number of nodules when compared to the wild-type. The mutation is described in various legumes, including *Medicago truncatula* Mt*_sunn_* [7], *Glycine max* Gm*_NARK_* [8], *Lotus japonicus* Lj*_HAR1_* [9], *Pisum sativum* Ps*_SYM29_* [10]. Under field conditions, the mutants show a comparatively poor performance [11]. This is attributed to the fact that the formation of the excessive nodules puts a metabolic burden on the plant and in addition the plant is not able to support the numerous nodules with sufficient assimilates. Nodules of supernodulating mutants are often of small size and low specific activity [12]. While various data convincingly show that photosynthesis is a crucial factor for the poor performance of supernodulating mutants under field conditions [11], it could thus far not be shown that optimal growth conditions and elevated CO_2_ assimilation can improve the activity of the excessive number of nodules of the mutant lines. The objective of this study was to clarify through diverse experimental approaches whether under optimal growth conditions the nodule activity is limited by assimilate supply. For that purpose, nodule activity was followed through nodule H_2_ evolution measurements while photosynthetic activity of the leaves was altered. In addition, the diversion of nodule activity during whole plant P-depletion from a fully nourished control was compared on *sunn* plants with plants of the wild-type (Jemalong A17). P deficiency is known to have a strong impact on leave CO_2_ assimilation [13].

## 2. Results and Discussion

### 2.1. The Effect of Increased Photosynthesis on the Specific Activity of the Nodules

The growth conditions for *M. truncatula* plants appear to be optimal in our system. Unlimited access to intensely aerated water (nutrient solution) is combined with a constant nutrient supply at optimal levels [14]. Additionally, the plants receive intensive light during a period of 16 h at optimal temperature (25 °C). The plants can reach up to 12 g dry matter (DM) during 8 weeks of growth in that system, depending solely on nitrogen fixation for N-nutrition (Ricardo A. Cabeza, data not shown). Although such data can only be compared reluctantly, this growth rate exceeds those found in the literature for nutrient solution or aeroponic growth of *M. truncatula* (e.g., [15]).

Increasing photosynthesis through elevated CO_2_ concentrations [16] or higher temperature around shoots [17] remained without a short-term reaction of nodule activity (Tables 1 and 2). Neither the per plant nitrogenase activity nor the relative efficiency of nitrogen fixation (EAC) could be affected through a 6 h elevated temperature of 10 degree nor through a three-fold increase in the concentration of CO_2_ around shoots for three weeks. At least the significant increased assimilate availability through elevated CO_2_ around the shoots should have shown an effect in case the nodules were assimilate limited. Assimilated carbon can reach the nodules within minutes [18–20].

Legumes are known to answer to long-term elevated CO_2_ with a concerted reaction. Per plant nitrogen fixation is adapted to increased demand largely by the formation of new nodules rather than increased specific activity of the existing nodules [21–23]. Consistent with these observations were the effect of long-term elevated carbon dioxide around shoots of the wild type (Table 3). In fact, the specific activity of the nodules in the wild-type plant was lower under elevated CO_2_ and remained unchanged in *sunn*. The formation of the excessive number of nodules in the mutant occurs in competition with root formation. According to our data, the carbon burden affects in particular root growth [24]. Our data furthermore suggest that elevated CO_2_ around shoots and thus increased CO_2_ assimilation partially rescue that detrimental effect on root growth. The fact that roots of *sunn* develop comparatively poorly might be part of the reason for the limited agronomical success of supernodulating mutants.

The amount of CO_2_ that is necessary to be pumped into the shoot compartment to keep the chosen threshold of CO_2_ concentration indicates the photosynthetic activity of the plants. Since the long-term experiment was performed in a greenhouse under natural light with shifting intensity, we cannot quantify the effect of the elevated CO_2_ concentration on CO_2_-assimilation precisely. Nevertheless, besides the fact that we observed increases in root and shoot dry matter, the higher influx into the shoot compartment with elevated CO_2_ was obvious, in particular under sunny conditions. Both the short- and long-term treatments that increased photosynthesis did not increase the specific activity of the *sunn* nodules. For legumes with a functioning AON and a regulated number of nodules, most experimental data indicate that assimilate supply to nodules is finely adapted rather than limiting for their activity. This opinion remained largely unchallenged since the comprehensive review of Vance and Heichel [22]. In addition, various studies on the carbon expenditure for driving nitrogenase activity (respired carbon by the nodules per unit reduced N) strengthened that view. For instance, most legume nodules under optimal and undisturbed conditions respire more carbon than needed when a most efficient respiration in terms of the avoidance of alternative respiration, activity of external NAD(P)H-ubiquinone oxidoreductases and uncoupling proteins is assumed and the relative efficiency of nitrogenase (electron allocation) is high [25]. The carbon efficiency of nitrogen fixation can be significantly increased (lower amount of oxidized C per reduced N) at the same plants, when the inner plant competition for assimilates increases due to pod formation (vegetative *vs*. reproductive growth) [26]. Consequently, under conditions of undisturbed growth legumes appear to use assimilates for driving nitrogenase in excess of what is necessary [25].

### 2.2. The Effect of Phosphorus Depletion on Nitrogen Fixation and Photosynthesis

**Figure 1.**
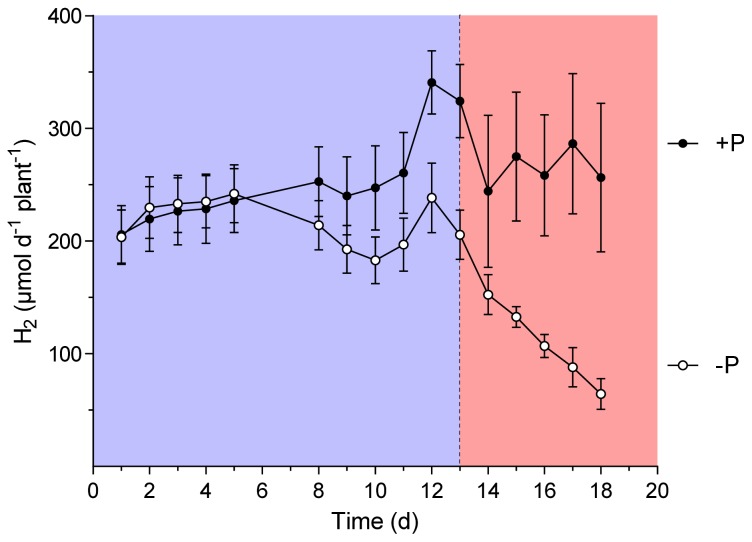
Apparent nitrogenase activity (ANA) of *M. truncatula* wild-type plants grown at sufficient P supply (+P) and during P depletion (−P). Data are given as means of six replicates ± SE. Each point represent the integral of the amount of H_2_ evolved measured every five minutes over a 24 h period. The dotted line indicates the point in time when treatments began to differ significantly in ANA (*t*-test, *p <* 0.05). The colors distinguish the period of significantly different ANA (reddish) from the period of P depletion but not yet different H_2_ evolution (blue).

**Figure 2.**
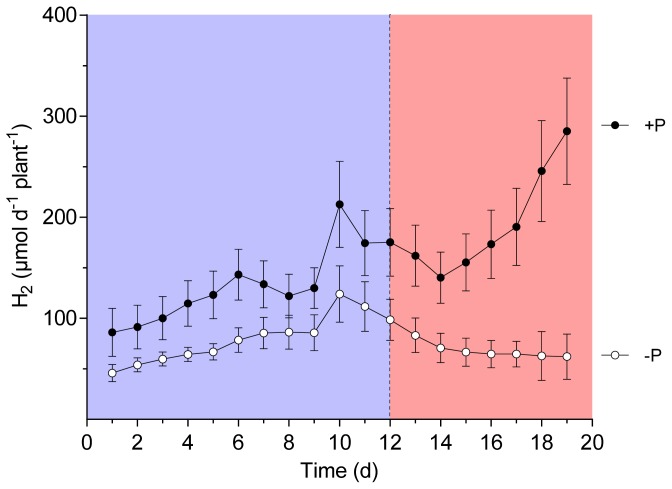
Apparent nitrogenase activity (ANA) of *M. truncatula sunn* plants grown at sufficient P supply (+P) and during P depletion (−P). Data are given as means of six replicates ± SE. Each point represent the integral of the amount of H_2_ evolved measured every five minutes over a 24 h period. The dotted line indicates the point in time when treatments began to differ significantly in ANA (*t*-test, *p <* 0.05). The colors distinguish the period of significantly different ANA (reddish) from the period of P depletion but not yet different H_2_ evolution (blue).

**Figure 3.**
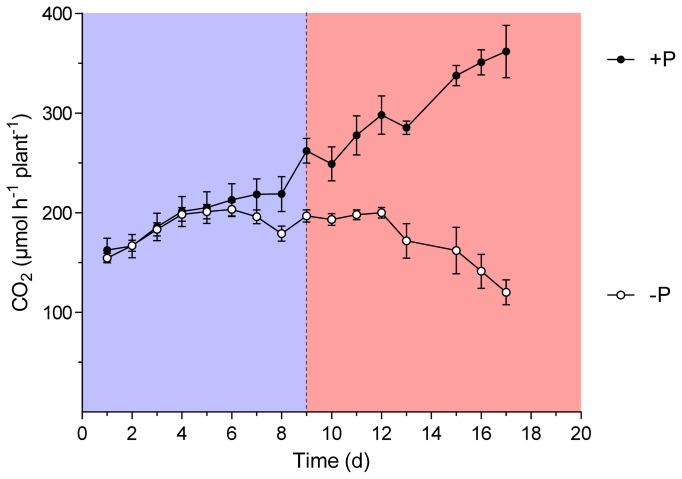
Root/nodule CO_2_ release of *M. truncatula* wild-type plants grown at sufficient P supply (+P) and during P depletion (−P). Data are given as means of six replicates ± SE. Each point represent the amount of CO_2_ evolved measured at 11 am. The dotted line indicates the point in time when treatments began to differ significantly (*t*-test, *p <* 0.05). The colors distinguish the period of significantly different ANA (reddish) from the period of P depletion but not yet different CO_2_ evolution (blue).

**Figure 4.**
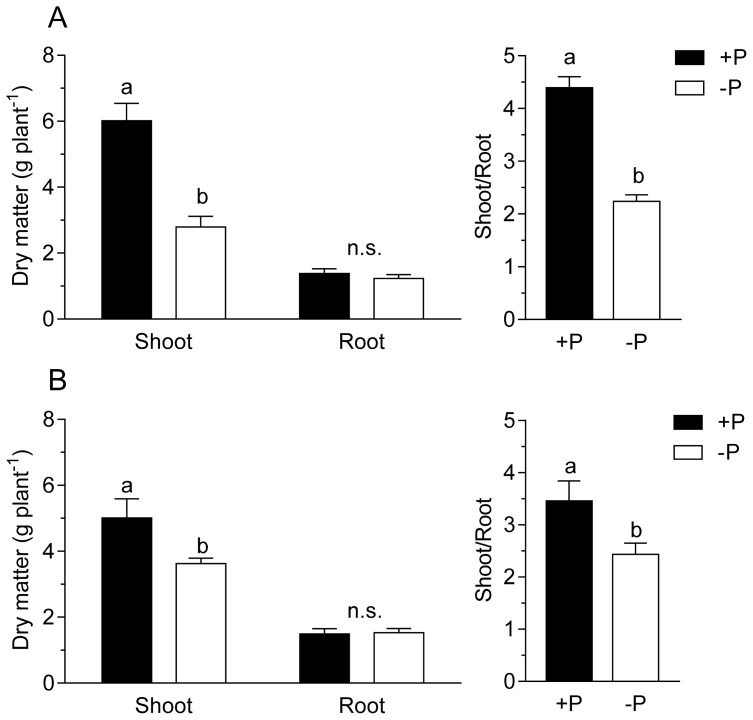
Plant dry matter and shoot/root ratio of (**A**) *sunn* and (**B**) wild-type plants at the end of the P-depletion experiment. +P stands for sufficient P supply and −P for a three-week P-depletion treatment. Data are means of six replicates ± SE. Lower case letters indicate a significant difference between treatments (*t*-test, *p <* 0.05, *n* = 6). n.s., not significant.

In a second experiment, a set of nitrogen fixing plants was exposed to P-free nutrient solution starting after 6 or 8 weeks of growth of wild-type and *sunn* plants, respectively. During the following 20 days, per plant H_2_ evolution of the nodules was continuously followed. Since P is of pivotal importance for leave CO_2_ assimilation and carbohydrate turnover, we hypothesized that an effect on nitrogen fixation should occur earlier in the supernodulating phenotype of the plant. The reasoning for the experiment was that it would form a supplemental treatment to the thus far performed experiments. In these experiments the effect of treatments that increased photosynthesis were studied. A P depletion would show the effect of impaired photosynthesis on nodule performance of the wild-type *vs. sunn* plants. The experiment was performed under optimal growth conditions other than limiting P in the treatment. In a similar approach, Hernandez *et al*. [27,28] showed that photosynthesis was increasingly impaired through P depletion. Figures 1 and 2 show the total amount of H_2_ evolution per plant and day as an integral of the continuously measured data. While a significant difference between the treatments was measured at 13 days of P depletion in the wild-type plants, this event occurred only one day earlier in the experiment with *sunn* plants. Root/nodule respiration measured daily at 11:00 am confirms the diversion of the treatment in the wild-type (Figure 3). Here the differences between the treatments reached already a significant value at day 9 after removal of P from the nutrient solution. Root/nodule CO_2_ release is closely related to nitrogen fixation activity [29]. Overall, the effect of whole plant P depletion on nitrogen fixation is not significantly more rapid in the *sunn* plants when compared to the wild type. In addition, while per plant nitrogen fixation of the *sunn* plants at the beginning of the experimental period was significantly lower when compared to the wild-type plants, the increase in the control plants was steeper than in *sunn* and the nodules of the P depletion treatment maintained constant activity per plant for almost as long as three weeks. By contrast, nodule activity of the wild-type plants in the P-depletion treatment decreased steadily during the second half of the experimental period. Dry matter formation at the end of the experiment confirms the strong effect of P-depletion (Figure 4). The reason for the higher shoot dry matter of the *sunn* plants compared the wild-type plants is in part a longer growth period before the beginning of the P-depletion treatment. The significantly higher shoot/root ratio of the mutant plants is a further point indicating that the excessive nodule growth and functioning is a burden for root development [24]. Taken together, the nitrogen fixation patterns during P depletion of *sunn vs.* wild-type plants render no indications that the nodule activity in *sunn* plants is assimilate-limited under the optimal growth conditions. However, transcriptomic studies revealed complex effect of P deficiency on nodule formation, development and functioning [28]. For instance, genes involved in nodule formation, symbiosome development and maintenance of nodule C- and N-fluxes are differentially expressed. Accordingly, the fact that wild-type and *sunn* did not differ in the response to P depletion might be the result of some other more direct impact on the nodules than assimilate supply.

**Figure 5.**
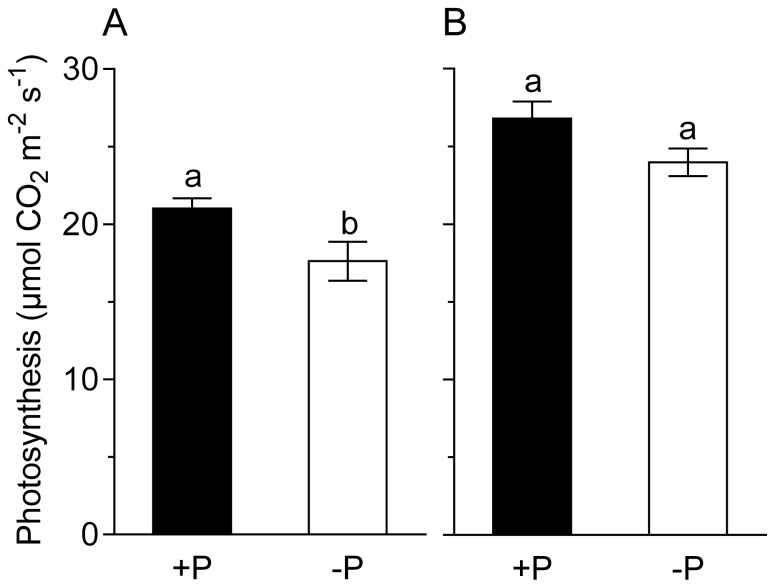
(**A**) Photosynthesis of *sunn* and (**B**) wild-type plants grown at sufficient P supply (+P) and after five to seven days of P depletion (−P). Data are given as means of six replicates ± SE. Lower case letters indicate a significant difference between treatments (*t*-test, *p <* 0.05, *n* = 6).

Specific CO_2_ assimilation of the leaves was measured at day 5 through 7 of the whole plant P depletion process (Figure 5). Specific CO_2_ assimilation was higher in the wild-type plants when compared to *sunn* plants. This confirms data of Voisin *et al*. [24]. In the wild-type plants the P depletion showed not yet a significant effect on specific CO_2_ assimilation, while it was reduced in the *sunn* plants by about 15%. The lower photosynthesis of *sunn* control plants might be a consequence of lower per plant nitrogen fixation in the mutant. It is a known fact that legumes, within limits, can adapt photosynthesis to the demand of the nodules [22]. The fact that the leaves of the *sunn* plants fix less CO_2_ is a further indication that a factor other than assimilation supply limited the nodule activity of *sunn* plants under our conditions. However, it cannot be ruled out that lower specific photosynthesis is a pleiotropic effect of the mutation in the *sunn*-gene [30].

### 2.3. Daily Patterns of Nodule H_2_ Evolution

**Figure 6.**
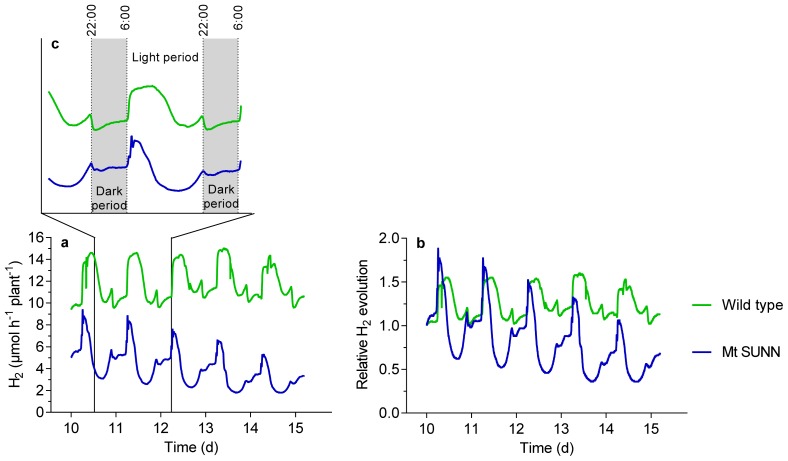
(**a**) Apparent nitrogenase activity (ANA), and (**b**) relative nitrogenase activity of *M. truncatula* wild-type and *sunn* plants at day 10 through 15 of the P-depletion treatment. The data in (**c**) show a period of 36 h with the dark periods indicated by grey color. Data are means of 6 replicates. Data for per plant H_2_ evolution were taken every five minutes.

Daily H_2_ evolution showed a clear pattern as shown in Figure 6. Figure 6c shows the pattern in greater detail in particular with an indication of the dark periods. The light was switched off at 10 pm. At that point in time a steep decline in activity occurred, which is largely temperature related. Nodule activity was subsequently maintained or even increased during the 8 h dark period. This was also the case in wild-type and *sunn* plants during the P-depletion experiment. When the light at 6 am in the morning was switched on, an again temperature related steep increase occurred, followed by slightly increasing activity in the morning and a first decline in the H_2_ evolution around noon. The subsequent decrease continued until about 5 pm. During the late afternoon, until 10 pm, the activity recovered. This pattern is typical for older plants. In young plants, a decline in nodule activity in the afternoon is almost undetectable but increases with the age of the plants. The overall daily rhythm of H_2_ evolution was not influenced by the P depletion treatment. However, there are clear differences between wild-type and *sunn* plants. While the overall pictures resemble (Figure 6a), the decline during the light period begins much earlier, about two to three hours after the light was switched on. A second clear difference is depicted in Figure 6b. Here, nodule activity at 3 am is set to 100% and the following time-course is shown relative to that value. The figure shows that the decline in the wild-type plants was about one-third of the peak activity at around noon, while in the *sunn* plants the decline was much stronger, accounting for about two-third of the peak activity.

Bergersen [31] pointed to the fact that biological processes, rather than being driven or down-regulated continuously, often oscillate around depleting pools. Assuming that the night and early morning nodule activity satisfies the N demand of the plant and the subsequent down regulation of the activity, is a result of a shoot factor (N-feedback [32,33]) that regularly slows down activity until a new demand emerges or the regulatory compound is used up. Such a mechanism would probably be confronted with higher amplitudes and the fine tuning to N demand would be much more difficult when the machinery (*i.e.*, the nodule number) that produces available nitrogen is much greater and its size and activity is not matched to N demand. Consequently, the slow performance of supernodulating mutants might as well be a result of the difficulties of the plants to fine tune the activity of a potentially strongly excessive machinery (nodules) to the demand of the plants and maybe also due to fluctuating availability of assimilates. The aberrant daily pattern of H_2_ evolution in *sunn* plants is an indication for that.

## 3. Experimental Section

### 3.1. Design of the Experiments

For the objective to determine whether nitrogen fixation of supernodulating *M. truncatula* plants would be assimilate limited under optimal growth conditions we performed three sets of experiments. In a first experiment short-term reactions of nodule activity to increased leave CO_2_ assimilation were monitored. In a second experiment, plants were exposed to elevated CO_2_ concentrations around the shoots for three weeks under greenhouse conditions with natural light (long-term experiment). Eventually, in a third experiment, nitrogen fixation was followed during a whole plant P depletion process. In this experiment the specific CO_2_ assimilation of the leaves was measured at 5 to 7 days after beginning the treatment.

### 3.2. Plant Growth

Seeds of *Medicago truncatula* (Gaertn.) cv. Jemalong A17 or *sunn* were submerged in H_2_SO_4_ (96%) for 5 min for chemical scarification, sterilized with 5% (*v*/*v*) sodium hypochlorite for 5 min and rinsed several times with deionized water. The seeds were subsequently kept at 4 °C for 12 h in darkness, submerged in tap water. The next step was a 2 to 4 day slight shaking of the submerged seeds at 25 °C and continuous light. When the seed had developed an about 20 mm long primary root, 20 plantlets each were transferred to small growth boxes (170 mm × 125 mm × 50 mm) filled with aerated nutrient solution. The seedlings were fixed through small x-shaped cuts in tape on the upper side of the growth boxes. The plants were grown for two weeks in these boxes in a growth chamber with a 16/8 h light/dark cycle at 25/20 °C, respectively. Light intensity at plant height was approximately 500 μmol·m^−2^·s^−1^. Immediately after transfer to the growth boxes, the seedlings were inoculated with 1 mL/box of a stationary *Sinorhizobium meliloti* (Sm) (102F51) YEM-culture, with an approximate cell density of 10^9^·mL^−1^. The Sm-strain induced good nodulation, with first eye-visible nodules after about 7 to 10 days. Wild-type plants developed only 2 to 5 visible nodules during the two-week growth in the growth boxes, while the *sunn* plants developed many. Sm 102F51 does not contain an uptake hydrogenase [34].

After two weeks, the plants were transferred to glass tubes, which allowed the separate measurement of root/nodule H_2_ and CO_2_ evolution. The system is described in Fischinger and Schulze [35]. We extended the set-up by connecting a group of six plants through the lower side of the glass tubes to a 20 L nutrient solution container. Thus through gravity, the nutrient solution level in the glass tubes was depending on the height of the container position and losses through plant transpiration could be adjusted by the addition of nutrient solution to the container, thereby not interfering with the measurements in the root/nodule compartment, in particular during the long-term experiments. In addition, a pump in the container drove a nutrient solution flow of about 10 mL·min^−1^ into the upper side of each individual glass tube. Each individual glass tube contained about 150 mL nutrient solution, and were turned over every 15 min. The nutrient solution in each glass tube was individually aerated with a gas stream of 200 mL·min^−1^ (N_2_/O_2_; 80/20; *v*/*v*). The nutrient solution contained: macronutrients (mM): K_2_SO_4_, 0.7; MgSO_4_, 0.5; CaCl_2_, 0.8; and micronutrients (μM): H_3_BO_3_, 4.0; Na_2_MoO_4_, 0.1; ZnSO_4_, 1.0; MnCl_2_, 2.0; CoCl_2_, 0.2; CuCl_2_, 1.0 and FeNaEDTA (ferric monosodium salt of ethylenediamine tetraacetic acid), 30. The pH was adjusted to 6.4 through KOH and buffered with 0.25 mM 2-(*N*-morpholino) ethane-sulfonic acid (MES). Phosphorus (P) was added daily as KH_2_PO_4_ to a concentration of 5 μM P. For the beginning of the P depletion treatment, the daily P application was stopped. During the first week after transfer to the nutrient solution, the solution was once adjusted to a 0.5 mM NH_4_^+^ concentration through the addition of NH_4_SO_4_. Low concentrations of ammonium support nodule formation in *M. truncatula* [36]. The nutrient solution was changed every week. During this procedure, the pump in the container was switched off and the backflow from the glass tubes to the container was blocked. In this way, the ongoing measurements in the root/nodule compartment were not affected. After the first week of growth in the glass tubes, the plants depended solely on N_2_ fixation for N nutrition.

### 3.3. Root/Nodule Gas Exchange Measurement

The system for measuring nodule H_2_ and CO_2_ evolution, including the determination of apparent nitrogenase activity (ANA), total nitrogenase activity (TNA), the calculation of the electron allocation coefficient (EAC) and of N_2_ fixation, is described in Fischinger and Schulze [37]. For a continuous, long-term measurement of H_2_ evolution, we extended the set-up through an efficient three-step air drying system for the airstream flowing out of the root/nodule compartment. Root/nodule CO_2_ evolution was measured daily at 11 am in the gas stream that was continuously analyzed for H_2_ concentration. The CO_2_ measurement was performed with a S151 CO_2_ analyzer (Qubit, Kingston, ON, Canada).

### 3.4. Elevated CO_2_ and Temperature around Shoots

For managing the atmosphere around shoots, a set of 12 plant shoots were enclosed in a plexiglass container. In these containers, temperature (10–35 °C), humidity (30%–60%) and CO_2_ concentration (0.005% to 100%) could be continuously regulated and maintained over weeks. The procedure is described in Schulze and Merbach [38]. In short: the air in the container was turned over by 4 fans pressing it through coolers that where supplied with water of adjustable temperature. In addition, the temperature was measured at plants height. A pump took a 200 mL airstream from around the shoots and pumped it through a CO_2_ analyzer. An automatic system switched two heaters inside the container or a low flow of CO_2_ into the container on when a threshold was undershot. The CO_2_ was supplied behind the fans so that it quickly mixed. Both systems were switched off when the threshold was reached again. In this way, the temperature could be increased from 20 to 30 °C within 5 min and the CO_2_ concentration from 400 to 1200 ppm within 12 min. The threshold could be kept with 1% and 4% over- and undershooting for temperature and CO_2_ concentration, respectively. The system was located in a climate chamber for the short-term experiments and in a greenhouse for the long-term experiment with elevated CO_2_.

### 3.5. Measurement of Specific Leave CO_2_ Assimilation

For measurement of specific CO_2_ assimilation, two leaflets were included in a small airtight compartment of the LI-6400XT portable photosynthesis measurement system (LI-COR, Lincoln, NE, USA). The measurements were performed on fully expanded leaves from 11 am through 3 pm at day 5 through 7 of the whole plants P depletion experiments. The surface area of the leaflets was determined through scanning.

## 4. Conclusions

Several results show that nitrogen fixation in the Mt*_sunn_* genotype is not limited by assimilate supply at optimal growth conditions. This is supported by the fact that neither short- nor long-term increased assimilate availability affected nodule specific activity neither in the wild-type plants nor in Mt*_sunn_*. In addition, whole plant P depletion did not show earlier or more intense effects on nitrogen fixation in the Mt*_sunn_* genotype when compared to the wild type. Eventually, specific photosynthesis was higher in the wild-type plants, which is not consistent with assimilate shortage of Mt*_sunn_* nitrogen fixation. A clear difference in the daily pattern of nitrogen fixation in Mt*_sunn_*, when compared to the wild-type, illustrates that the fine tuning of the excessive nitrogen fixation capacity of the mutant is difficult for the plant. As a conclusion, the poor performance of the mutant under field conditions might in addition to the high assimilate burden of the supernodulating phenotype be explained by difficulties in regulating the activity of the large number of nodules on whole plant level.

## Figures and Tables

**Table 1.** H_2_ evolution of *M. truncatula* nodules after short-term increase of the temperature around shoots. Data are given as means of 10 replicates. The electron allocation coefficient (EAC, relative efficiency of nitrogenase) was measured at the beginning and the end of the experiment Data were compared statistically (within a column) between the points in time of the analysis but showed no significant differences (*Tukey*’s test, *p <* 0.05, *n =* 10; comparison of the EAC by the *t*-test, *p <* 0.05, *n =* 10).

Growth conditions	H_2_ evolution	

	[μmol H_2_·h^−1^·plant^−1^]	EAC
20 °C/400 ppm CO_2_ around shoots	4.68	0.59
20 °C around nodules		
**1 h**		
30 °C/400 ppm CO_2_ around shoots	4.64	n.d.
20 °C around nodules		
**2 h**		
30 °C/400 ppm CO_2_ around shoots	4.64	n.d.
20 °C around nodules		
**6 h**		
30 °C/400 ppm CO_2_ around shoots	4.73	0.61
20 °C around nodules		

**Table 2.** H_2_ evolution of *M. truncatula* nodules after short-term increase of the CO_2_ concentration around shoots. Data are given as means of 8 replicates. The electron allocation coefficient (EAC, relative efficiency of nitrogenase) was measured at the beginning and the end of the experiment. Data were compared statistically (within a column) between the points in time of the analysis but showed no significant differences (*Tukey*’s test, *p <* 0.05, *n =* 10; comparison of the EAC by the *t*-test, *p <* 0.05, *n =* 10).

Growth conditions	H_2_ evolution	

	[μmol H_2_·h^−1^·plant^−1^]	EAC
25 °C/400 ppm CO_2_ around shoots	5.27	0.63
20 °C around nodules		
**1 h**		
25 °C/1200 ppm CO_2_ around shoots	5.31	n.d.
20 °C around nodules		
**2 h**		
25 °C/1200 ppm CO_2_ around shoots	5.35	n.d.
20 °C around nodules		
**6 h**		
25 °C/1200 ppm CO_2_ around shoots	5.38	n.d.
20 °C around nodules		
**24 h**		
25 °C/1200 ppm CO_2_ around shoots	5.24	0.58
20 °C around nodules		

**Table 3.** Growth and nitrogen fixation of wild-type and *sunn M. truncatula* plants. Data are given per plant as means of 6 replicates. Specific N_2_-fixation was calculated from the N increment in plants (N-free nutrient solution) and the number of nodules at the end of the experimental period.

Parameter	Wild-type		Mt*_sunn_*	
	
	400 ppm CO_2_	1200 ppm CO_2_	400 ppm CO_2_	1200 ppm CO_2_
Shoot [g DM]	3.4	4.1 *	2.7	3.4 *
Root/Nodules [g DM]	1	1.7	0.6	1.3 *
Nodule Number per plant	186	287	798	1004
Specific N_2_ fixation [μg N·day^−1^·nodule^−1^]	1.45	0.93 *	0.26	0.31

* indicates a statistically significant difference when compared to plants grown at 400 ppm CO_2_ around shoots (*t*-test, *p <* 0.05, *n* = 6).

Plants were grown for 8 weeks at ambient CO_2_. Subsequently the treatment and control conditions were maintained for three weeks. The experiment was done under greenhouse conditions with natural light and the plants enclosed in a plexiglass chamber with regulated atmosphere.

## References

[1] Larue T.A., Patterson T.G. (1981). How much nitrogen do legumes fix?. Adv. Agron.

[2] Schulze J., Adgo E., Merbach W. (1999). Carbon costs associated with N_2_ fixation in *Vicia faba* L. and *Pisum sativum* L. over a 14-day period. Plant Biol.

[3] Cabeza R., Koester B., Liese R., Lingner A., Baumgarten V., Dirks J., Salinas-Riester G., Pommerenke C., Dittert K., Schulze J. (2014). An RNA sequencing transcriptome analysis reveals novel insights into molecular aspects of the nitrate impact on the nodule activity of*Medicago truncatula*. Plant Physiol.

[4] Ferguson B.J., Indrasumunar A., Hayashi S., Lin M.H., Lin Y.H., Reid D.E., Gresshoff P.M. (2010). Molecular analysis of legume nodule development and autoregulation. J. Integr. Plant Biol.

[5] Searle I.R., Men A.E., Laniya T.S., Buzas D.M., Iturbe-Ormaetxe I., Carroll B.J., Gresshoff P.M. (2003). Long-distance signaling in nodulation directed by a CLAVATA1-like receptor kinase. Science.

[6] Lin Y.-H., Ferguson B.J., Kereszt A., Gresshoff P.M. (2010). Suppression of hypernodulation in soybean by a leaf-extracted, NARK- and Nod factor-dependent, low molecular mass fraction. New Phytol.

[7] Schnabel E., Journet E.P., de Carvalho-Niebel F., Duc G., Frugoli J. (2005). The *Medicago truncatula* SUNN gene encodes a CLV1-like leucine-rich repeat receptor kinase that regulates nodule number and root length. Plant Mol. Biol.

[8] Carroll B.J., McNeil D.L., Gresshoff P.M.A. (1985). Supernodulation and nitrate-tolerant symbiotic (Nts) soybean mutant. Plant Physiol.

[9] Nishimura R., Hayashi M., Wu G.J., Kouchi H., Imaizumi-Anraku H., Murakami Y., Kawasaki S., Akao S., Ohmori M., Nagasawa M. (2002). HAR1 mediates systemic regulation of symbiotic organ development. Nature.

[10] Krusell L., Madsen L.H., Sato S., Aubert G., Genua A., Szczyglowski K., Duc G., Kaneko T., Tabata S., de Bruijn F. (2002). Shoot control of root development and nodulation is mediated by a receptor-like kinase. Nature.

[11] Novak K. (2010). On the efficiency of legume supernodulating mutants. Ann. Appl. Biol.

[12] Buttery B.R., Park S.J. (1990). Effects of nitrogen, inoculation and grafting on expression of supernodulation in a mutant of *Phaseolus-vulgaris* L. Can. J. Plant Sci.

[13] Fredeen A.L., Raab T.K., Rao I.M., Terry N. (1990). Effects of phosphorus nutrition on photosynthesis in *Glycine max* (L.) Merr. Planta.

[14] Sulieman S., van Ha C., Schulze J., Tran L.-S.P. (2013). Growth and nodulation of symbiotic *Medicago truncatula* at different levels of phosphorus availability. J. Exp. Bot.

[15] Tang C., Hinsinger P., Drevon J.J., Jaillard B. (2001). Phosphorus deficiency impairs early nodule functioning and enhances proton release in roots of *Medicago truncatula* L. Ann. Bot.

[16] Makino A., Mae T. (1999). Photosynthesis and plant growth at elevated levels of CO_2_. Plant Cell Physiol.

[17] Pastenes C., Horton P. (1996). Effect of high temperature on photosynthesis in beans. 2. CO_2_ assimilation and metabolite contents. Plant Physiol.

[18] Voisin A.S., Salon C., Jeudy C., Warembourg F.R. (2003). Root and nodule growth in *Pisum sativum* L. in relation to photosynthesis: analysis using ^13^C-labelling. Ann. Bot.

[19] Kouchi H., Akao S., Yoneyama T. (1986). Respiratory utilization of C-13-labeled photosynthate in nodulated root systems of soybean plants. J. Exp. Bot.

[20] Kouchi H., Yoneyama T., Akao S. (1986). Compartmental analysis of the partitioning of photo-assimilated carbon in nodulated soybean plants during the light period. J. Exp. Bot.

[21] Rogers A., Ainsworth E.A., Leakey A.D.B. (2009). Will Elevated carbon dioxide concentration amplify the benefits of nitrogen fixation in legumes?. Plant Physiol.

[22] Vance C.P., Heichel G.H. (1991). Carbon in N_2_ fixation-limitation or exquisite adaptation. Annu. Rev. Plant Phys.

[23] Cen Y.P., Layzell D.B. (2004). Does oxygen limit nitrogenase activity in soybean exposed to elevated CO_2_?. Plant Cell Environ.

[24] Voisin A.S., Cazenave A.B., Duc G., Salon C. (2013). Pea nodule gradients explain C nutrition and depressed growth phenotype of hypernodulating mutants. Agron. Sustain. Dev.

[25] Schulze J. (2004). How are nitrogen fixation rates regulated in legumes?. J. Plant Nutr. Soil Sci.

[26] Adgo E., Schulze J. (2002). Nitrogen fixation and assimilation efficiency in Ethiopian and German pea varieties. Plant Soil.

[27] Hernandez G., Ramirez M., Valdes-Lopez O., Tesfaye M., Graham M.A., Czechowski T., Schlereth A., Wandrey M., Erban A., Cheung F. (2007). Phosphorus stress in common bean: Root transcript and metabolic responses. Plant Physiol.

[28] Hernández G., Valdés-López O., Ramírez M., Goffard N., Weiller G., Aparicio-Fabre R., Fuentes S.I., Erban A., Kopka J., Udvardi M.K. (2009). Global changes in the transcript and metabolic profiles during symbiotic nitrogen fixation in phosphorus-stressed common bean plants. Plant Physiol.

[29] Fischinger S.A., Schulze J. (2010). The importance of nodule CO_2_ fixation for the efficiency of symbiotic nitrogen fixation in pea at vegetative growth and during pod formation. J. Exp. Bot.

[30] Burstin J., Marget P., Huart M., Moessner A., Mangin B., Duchene C., Desprez B., Munier-Jolain N., Duc G. (2007). Developmental genes have pleiotropic effects on plant morphology and source capacity, eventually impacting on seed protein content and productivity in pea. Plant Physiol.

[31] Bergersen F.J. (1997). Regulation of nitrogen fixation in infected cells of leguminous root nodules in relation to O_2_ supply. Plant Soil.

[32] Sulieman S., Fischinger S.A., Gresshoff P.M., Schulze J. (2010). Asparagine as a major factor in the N-feedback regulation of N_2_ fixation in*Medicago truncatula*. Physiol. Plant.

[33] Sulieman S., Schulze J. (2010). Phloem-derived gamma-aminobutyric acid (GABA) is involved in upregulating nodule N_2_ fixation efficiency in the model legume*Medicago truncatula*. Plant Cell Environ.

[34] Blumenthal J.M., Russelle M.P., Vance C.P. (1997). Nitrogenase activity is affected by reduced partial pressures of N_2_ and NO_3_^−^. Plant Physiol.

[35] Fischinger S.A., Hristozkova M., Mainassara Z.-A., Schulze J. (2010). Elevated CO_2_ concentration around alfalfa nodules increases N_2_ fixation. J. Exp. Bot.

[36] Fei H., Vessey J.K. (2009). Stimulation of nodulation in *Medicago truncatula* by low concentrations of ammonium: Quantitative reverse transcription PCR analysis of selected genes. Physiol. Plant.

[37] Fischinger S.A., Schulze J. (2010). The argon-induced decline in nitrogenase activity commences before the beginning of a decline in nodule oxygen uptake. J. Plant Physiol.

[38] Schulze J., Merbach W.F. (2008). Nitrogen rhizodeposition of young wheat plants under elevated CO_2_ and drought stress. Biol. Fert Soils.

